# Laryngeal Mycosis in an Immunocompetent Patient: A Case Report

**DOI:** 10.7759/cureus.54241

**Published:** 2024-02-15

**Authors:** Rinor Ajeti

**Affiliations:** 1 Otolaryngology - Head and Neck Surgery, University of Pristina, Ferizaj, ALB; 2 Otolaryngology, RINO Clinic, Ferizaj, ALB

**Keywords:** dysphagia, hoarseness, immunocompetence, antifungal treatment, laryngitis

## Abstract

Laryngeal mycosis, a condition often overlooked in systemically immunocompetent individuals, requires heightened clinical vigilance for accurate diagnosis. The disease mimics symptoms of other laryngeal conditions such as gastroesophageal reflux, granulomatous disease, keratosis, and glottic malignancies, with risk factors including prolonged use of antibiotics, inhaled steroids, and smoking. Clinically, it presents with variable symptoms including hoarseness, and occasionally pain, dysphagia, and odynophagia. Diagnosis involves the observation of hyperkeratosis, notably when intraepithelial neutrophils are present, prompting further investigation for fungal elements through specialized staining. Effective management encompasses prolonged systemic antifungal treatment and the elimination of predisposing factors to prevent recurrence or treatment failure. Despite its potential to mimic a range of laryngeal diseases, laryngeal mycosis remains a less considered differential diagnosis. This is compounded by the commonality of risk factors in the general population, including prolonged antibiotic use, inhaled steroid therapy, and smoking habits, which may predispose individuals to fungal infections of the larynx. Furthermore, the necessity for a high index of suspicion and specialized diagnostic techniques, such as the identification of hyperkeratosis with intraepithelial neutrophils through biopsy and specialized staining for fungal elements, underscores the complexity of diagnosing this condition. The rationale for documenting this case report is multifaceted, primarily focusing on the fact that laryngeal mycosis is rare among immunocompetent patients leading to under-recognition of laryngeal mycosis in systemically immunocompetent individuals and the diagnostic challenges it presents. Additionally, the documentation seeks to emphasize the critical need for comprehensive treatment approaches, including prolonged systemic antifungal therapy and the identification and elimination of predisposing factors, to ensure effective management and prevent recurrence.

## Introduction

Fungal laryngitis is an infrequent occurrence among immunocompetent patients, and it can often manifest with non-specific clinical symptoms resembling leukoplakia. Unless healthcare providers consider the possibility of fungal laryngitis, there is a risk of misdiagnosis, leading to inappropriate treatment and prolonged patient discomfort.

In cases where fungal laryngitis is under consideration, employing video laryngoscopy for laryngeal examination can uncover various indicative signs such as redness (erythema), swelling (edema), thickened skin patches (hyperkeratosis), sticky white plaques, minor surface wounds (shallow ulcerations), and the emergence of gray or white false membranes enveloping the vocal cords [1]

To ensure an accurate diagnosis, a thorough examination of the oral cavity and oropharynx should be conducted to rule out any fungal infections [2]. Additionally, fiberoptic nasopharyngolaryngoscopy is essential to pinpoint the precise laryngeal pathology. Factors including the utilization of inhaled steroids, the presence of laryngopharyngeal reflux disease, and extended antibiotic therapy can heighten the risk of developing fungal laryngitis, potentially resulting in symptoms like voice hoarseness [2].

## Case presentation

A male patient, aged 45, presented with a complaint of voice hoarseness and swallowing difficulties persisting for a period of two months. Notably, the patient had a 20-year history of smoking, did not engage in alcohol consumption, had no history of asthma or the use of inhaled steroids, and tested positive for *Helicobacter pylori*.

Three months ago, the patient had weakening of muscle strength, muscle pain, difficulty in walking, general fatigue, and some changes in the skin and was diagnosed with dermatomyositis. Subsequently, he was admitted to the rheumatology department at a university clinical center for an extended one-month duration. During this admission, the patient experienced symptoms including fever, weight loss, fatigue, redness of the eyes, sore throat, and decreased appetite. Histopathological examination and gastroscopy revealed esophageal candidiasis and he was treated with antibiotics for *H. pylori* and nystatin suspension for candidiasis for seven days. He was discharged from the hospital with a diagnosis of hiatal hernia, candidal esophagitis, hyperferritinemia, and dermatomyositis, without improvement of voice or dysphagia.

It was two days after discharge that he came to our ENT clinic for evaluation. Video laryngoscopy was done which revealed elevated whitish plaques on the membranous vocal folds, primarily on the left side, numerous white patches located at the junction of the anterior and posterior commissure of the right vocal cord, as well as on the posterior pharyngeal wall, right aryepiglottic fold, and pyriform sinus, and redness (erythema) surrounding the vocal cords and the anterior wall of the epiglottis (Video 1). Abundant secretions were present throughout the entire larynx.

**Video 1:** Video endoscopy prior to treatment revealed whitish plaques on the membranous vocal folds, particularly on the left side, and numerous white patches at the junction of the anterior and posterior commissures of the right vocal cord, as well as on the posterior pharyngeal wall, the right aryepiglottic fold, the pyriform sinus, the anterior wall of the epiglottis, and the surrounding area of the vocal cords displayed erythema.

The patient was advised about the necessity of further investigation through a biopsy if their symptoms did not improve. Based on clinical features, gastroenterologist reports, and the video laryngoscopy, a preliminary diagnosis of esophageal candidiasis was made. Consequently, oral itraconazole treatment was initiated at a dosage of 100 mg twice daily for three weeks [3]. Remarkably, the patient experienced a significant improvement in their symptoms. A follow-up examination of the larynx at the end of the three weeks revealed complete resolution of both symptoms and signs (Video 2). 

**Video 2:** Video endoscopy after treatment showed complete resolution of symptoms and signs after three weeks

## Discussion

Fungal laryngitis is a relatively uncommon but significant medical condition that affects the larynx, which is the part of the throat containing the vocal cords. It occurs when fungal pathogens, such as Candida species [3], invade the laryngeal tissues, leading to a range of symptoms and potential complications. This condition is often associated with certain predisposing factors that can weaken the immune system or disrupt the mucosal barrier in the throat .

Pathologists frequently do not utilize fungal staining techniques in laryngeal biopsy assessments. In some cases, a re-examination of the original tissue with the appropriate staining methods may be necessary. The predominant fungal culprit is *Candida albicans*, although less common forms such as *Blastomyces*, *Histoplasma*, and *Aspergillus* can also be encountered [4]. For cases that are resistant to standard treatment or involve extensive tissue invasion, intravenous amphotericin B is employed as a therapeutic option [5].

Another set of factors that increase the risk of fungal laryngitis involves changes in the mucosal barrier. Radiotherapy to the head and neck region can damage the protective lining of the throat, making it easier for fungi to colonize and cause infection. Additionally, the use of inhaled corticosteroids, often prescribed for respiratory conditions, can disrupt the local immune defenses in the throat. Gastroesophageal reflux disorder (GERD) is another contributing factor, as it can introduce stomach acid and digestive enzymes into the throat, creating an environment conducive to fungal growth [6]. Trauma to the larynx, such as that caused by prolonged intubation during medical procedures, can also increase the risk of fungal laryngitis. The irritation and damage to the laryngeal tissues create opportunities for fungal pathogens to take hold.

One of the key predisposing factors is a compromised immune system. Individuals with conditions like diabetes, immunodeficiency disorders, or those undergoing immunosuppressive treatments such as chemotherapy or long-term corticosteroid use, are more susceptible to fungal laryngitis. The weakened immune response allows fungal organisms to proliferate in the laryngeal tissues, causing symptoms like hoarseness [7], difficulty swallowing, and throat discomfort. Furthermore, smoking has been associated with an increased risk of fungal laryngitis [7]. The toxins and irritants in cigarette smoke can compromise the health of the mucosal lining of the throat, making it more susceptible to fungal infections.

Diagnosis of fungal laryngitis typically involves laryngoscopy, where a flexible or rigid endoscope is used to visualize the larynx and assess the extent of fungal involvement. Treatment usually consists of antifungal medications, either in the form of oral medications or topical treatments applied directly to the larynx. Addressing any underlying predisposing factors, such as managing diabetes or discontinuing immunosuppressive medications, is also essential for a successful recovery.

In summary, fungal laryngitis is a condition that primarily affects individuals with weakened immune systems or those with disrupted mucosal barriers in the throat. Recognizing and addressing the underlying predisposing factors, along with appropriate antifungal treatment, are crucial steps in managing this condition and ensuring a favorable outcome for affected individuals.

The presence of hyperkeratosis [2], especially when accompanied by intraepithelial neutrophils, should prompt a biopsy to investigate the potential presence of fungal elements using specialized staining techniques. Patients typically seek medical attention due to symptoms such as hoarseness and dysphagia. It's important to note that laryngeal mycosis is typically a secondary infection, whereas primary laryngeal infections are predominantly observed in individuals with compromised immune systems.

The patient in the reported case was administered Itraconazole 100 mg twice a day for three weeks and significant improvement with complete resolution of signs and symptoms [3].

## Conclusions

Diagnosing fungal laryngitis in immunocompetent patients can be challenging due to its relatively rare occurrence in this population. As a result, medical professionals need to maintain a high level of suspicion when assessing patients with relevant symptoms or risk factors. When hyperkeratosis, which is the thickening of the epithelial layer of the larynx, is present, it raises strong suspicion for fungal laryngitis. However, a definitive diagnosis requires a biopsy of the affected tissue. This biopsy is essential as it allows for the examination of tissue samples under a microscope, which may reveal the presence of fungal elements and confirm the diagnosis.

Once fungal laryngitis is diagnosed, treatment involves a prolonged course of systemic antifungal medication. This typically means taking antifungal drugs by mouth to target the infection throughout the body. Additionally, it's essential to identify and address any predisposing factors that may have contributed to the development of the infection. For instance, if the patient has conditions or habits such as diabetes, smoking, or medication use that can weaken the immune system or disrupt the mucosal barrier, these factors should be managed or modified to prevent recurrence. Diagnosing fungal laryngitis in immunocompetent patients requires a vigilant and suspicious mindset, especially when hyperkeratosis is present. A biopsy is crucial to confirm the diagnosis, and treatment involves a prolonged course of systemic antifungal medication, along with addressing any underlying predisposing factors to ensure a successful recovery.
